# Long COVID after breakthrough SARS-CoV-2 infection

**DOI:** 10.1038/s41591-022-01840-0

**Published:** 2022-05-25

**Authors:** Ziyad Al-Aly, Benjamin Bowe, Yan Xie

**Affiliations:** 1 Clinical Epidemiology Center, Research and Development Service, VA Saint Louis Health Care System, St. Louis, MO USA; 2 Veterans Research and Education Foundation of Saint Louis, St. Louis, MO USA; 3 Department of Medicine, Washington University School of Medicine, St. Louis, MO USA; 4 Nephrology Section, Medicine Service, VA Saint Louis Health Care System, St. Louis, MO USA; 5 Institute for Public Health, Washington University in Saint Louis, St. Louis, MO USA; 6 Department of Epidemiology and Biostatistics, College for Public Health and Social Justice, Saint Louis University, St. Louis, MO USA

**Keywords:** SARS-CoV-2, Diseases, Viral infection, Vaccines, Vaccines

## Abstract

The post-acute sequelae of severe acute respiratory syndrome coronavirus 2 (SARS-CoV-2) infection—also referred to as Long COVID—have been described, but whether breakthrough SARS-CoV-2 infection (BTI) in vaccinated people results in post-acute sequelae is not clear. In this study, we used the US Department of Veterans Affairs national healthcare databases to build a cohort of 33,940 individuals with BTI and several controls of people without evidence of SARS-CoV-2 infection, including contemporary (*n* = 4,983,491), historical (*n* = 5,785,273) and vaccinated (*n* = 2,566,369) controls. At 6 months after infection, we show that, beyond the first 30 days of illness, compared to contemporary controls, people with BTI exhibited a higher risk of death (hazard ratio (HR) = 1.75, 95% confidence interval (CI): 1.59, 1.93) and incident post-acute sequelae (HR = 1.50, 95% CI: 1.46, 1.54), including cardiovascular, coagulation and hematologic, gastrointestinal, kidney, mental health, metabolic, musculoskeletal and neurologic disorders. The results were consistent in comparisons versus the historical and vaccinated controls. Compared to people with SARS-CoV-2 infection who were not previously vaccinated (*n* = 113,474), people with BTI exhibited lower risks of death (HR = 0.66, 95% CI: 0.58, 0.74) and incident post-acute sequelae (HR = 0.85, 95% CI: 0.82, 0.89). Altogether, the findings suggest that vaccination before infection confers only partial protection in the post-acute phase of the disease; hence, reliance on it as a sole mitigation strategy may not optimally reduce long-term health consequences of SARS-CoV-2 infection. The findings emphasize the need for continued optimization of strategies for primary prevention of BTI and will guide development of post-acute care pathways for people with BTI.

## Abstract (web summary)

A new analysis using the US Department of Veterans Affairs national healthcare databases demonstrates that Long COVID can occur after breakthrough SARS-CoV-2 infection; however, the risk of death attributable to COVID and incidence of post-acute sequelae were substantially reduced (but not fully eliminated) compared to unvaccinated individuals.

## Main

The post-acute sequelae of SARS-CoV-2 infection—also referred to as Long COVID—have been characterized^1^. Increasingly, vaccinated individuals are being diagnosed with COVID-19 as a result of breakthrough SARS-CoV-2 infection (BTI)^2,3^. Whether people with BTI experience post-acute sequelae is not clear. Addressing this knowledge gap is important to guide public health policy and post-acute COVID-19 care strategies.

Here we leverage the breadth and depth of the electronic healthcare databases of the US Department of Veterans Affairs to address the question of whether people with BTI develop post-acute sequelae. We characterize the risks and 6-month burdens of a panel of prespecified outcomes in a cohort of people who experienced BTI after completion of vaccination in the overall cohort and by care setting of the acute phase of the disease (that is, whether people were not hospitalized, hospitalized or admitted to an intensive care unit (ICU) during the first 30 days after a positive test). We then undertake a comparative evaluation of the magnitude of risk in people with BTI versus those with SARS-CoV-2 infection and no prior vaccination and, separately, hospitalized people with BTI versus those hospitalized with seasonal influenza.

## Results

### Post-acute sequelae in BTI versus controls without SARS-CoV-2 infection

There were 33,940 and 4,983,491 participants in the BTI group and a contemporary control group of users of the Veterans Health Administration from 1 January 2021 to 31 October 2021 with no record of a positive SARS-CoV-2 test, respectively. BTI participants had a positive SARS-CoV-2 test with prior record of a complete vaccination defined following Centers for Disease Control and Prevention (CDC) guidelines at 14 days after first Janssen (Johnson & Johnson)(Ad26.COV2.S) vaccination and 14 days after second Pfizer-BioNTech (BNT162b2) or Moderna (mRNA-1273) vaccination. The demographic and health characteristics of the BTI and the control groups before and after weighting are presented in Supplementary Tables 1–4. During the enrollment period, the overall rate of BTI within those fully vaccinated was 10.60 (95% CI: 10.52, 10.70) per 1,000 persons at 6 months; rates of breakthrough by vaccine type are presented in Supplementary Data Table 1.

For all analyses, we provide two measures of risk: (1) we estimated the adjusted HRs of a set of incident prespecified outcomes in people with BTI versus the control group; and (2) we estimated the adjusted excess burden of each outcome due to BTI per 1,000 persons 6 months after a positive SARS-CoV-2 test on the basis of the difference between the estimated incidence rate in individuals with BTI and the control group. Assessment of standardized mean differences of participant characteristics (from data domains including diagnoses, medications and laboratory test results) after application of weighting showed that they are well-balanced in each analysis of incident outcomes (Supplementary Fig. 1).

**Fig. 1:**
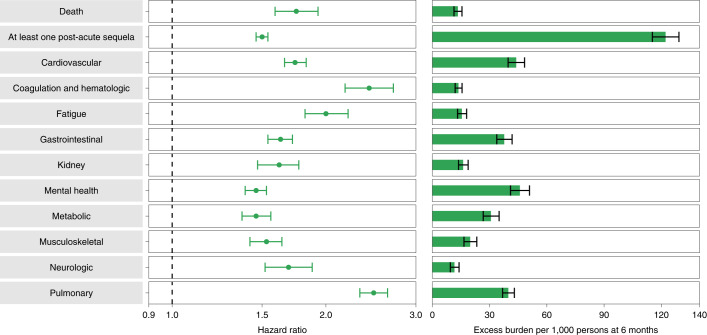
Risk and 6-month excess burden of post-acute sequelae in people with BTI compared to the contemporary control group. Risk and 6-month excess burden of death, at least one post-acute sequela and post-acute sequelae by organ system are plotted. Incident outcomes were assessed from 30 days after the positive SARS-CoV-2 test to the end of follow-up. Results are in comparison of BTI (*n* = 33,940) to the contemporary control group that consisted of those with no record of a positive SARS-CoV-2 test (*n* = 4,983,491). Adjusted HRs (dots) and 95% CIs (error bars) are presented, as are estimated excess burden (bars) and 95% CIs (error bars). Burdens are presented per 1,000 persons at 6 months of follow-up.

Compared to the contemporary control group, people who survived the first 30 days of BTI exhibited an increased risk of death (HR = 1.75, 95% CI: 1.59, 1.93) and excess burden of death estimated at 13.36 (95% CI: 11.36, 15.55) per 1,000 persons with BTI at 6 months; all burden estimates represent excess burden and are given per 1,000 persons with BTI at 6 months (Fig. 1). People with BTI also had an increased risk of having at least one post-acute sequela of SARS-CoV-2 (PASC) (HR = 1.50, 95% CI: 1.46, 1.54; burden of 122.22, 95% CI: 115.31, 129.24) (Supplementary Table 5).

**Extended Data Fig. 1:**
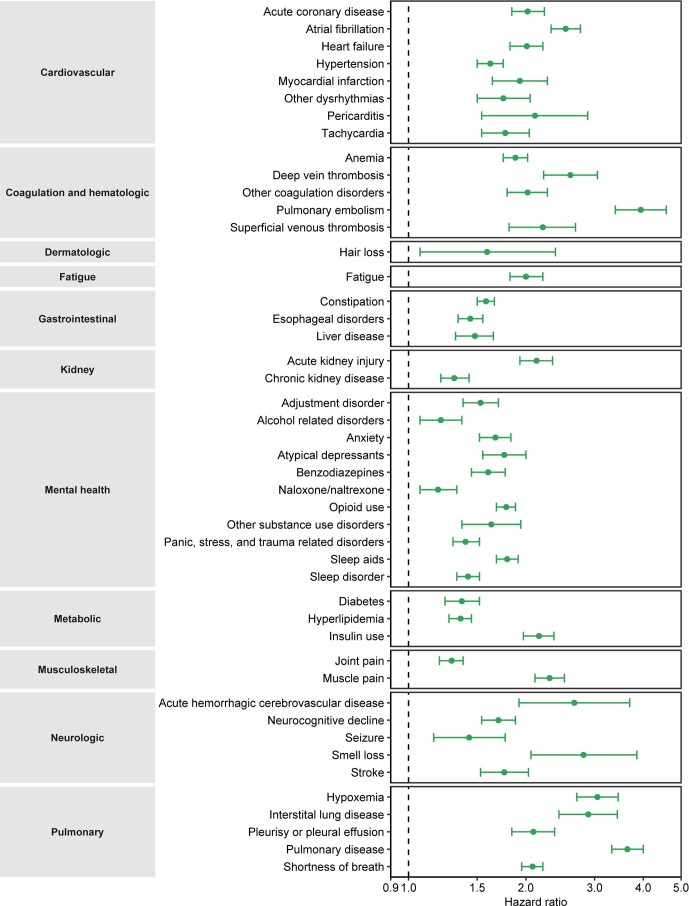
Risk of post-acute sequelae in people with breakthrough SARS-CoV-2 infection compared to the contemporary control group. Incident outcomes were assessed from 30 days after the positive SARS-CoV-2 test to end of follow-up. Results are in comparison of breakthrough SARS-CoV-2 infection (n = 33,940) to the contemporary control group that consisted of those with no record of a positive SARS-CoV-2 test (n = 4,983,491). Adjusted hazard ratios (dots) and 95% confidence intervals (error bars) are presented. Myocarditis (hazard ratio: 1.99; 95% confidence intervals: 0.47, 8.40) was not plotted to remove excess x-axis scaling.

Compared to the control group, 30-day survivors of BTI exhibited increased risk of post-acute sequelae in the pulmonary (HR = 2.48 (2.33, 2.64); burden of 39.82 (36.83, 42.99)) and several extrapulmonary organ systems, including cardiovascular disorders (HR = 1.74 (1.66, 1.83); burden of 43.94 (39.72, 48.35)), coagulation and hematologic disorders (HR = 2.43 (2.18, 2.71); burden of 13.66 (11.95, 15.56)), fatigue (HR = 2.00 (1.82, 2.21); burden of 15.47 (13.21, 17.96)), gastrointestinal disorders (HR = 1.63 (1.54, 1.72); burden of 37.68 (33.76, 41.80)), kidney disorders (HR = 1.62 (1.47, 1.77); burden of 16.12 (13.72, 18.74)), mental health disorders (HR = 1.46 (1.39, 1.53); burden of 45.85 (40.97, 50.92)), metabolic disorders (HR = 1.46 (1.37, 1.56); burden of 30.70 (26.65, 35.00)), musculoskeletal disorders (HR = 1.53 (1.42, 1.64); burden of 19.81 (16.56, 23.31)) and neurologic disorders (HR = 1.69 (1.52, 1.88); burden of 11.60 (9.43, 14.01)). Risk and excess burden of each individual sequela and by organ system are provided in Extended Data Fig. 1 (Supplementary Table 6) and Fig. 1 (Supplementary Table 5), respectively.

**Extended Data Fig. 2:**
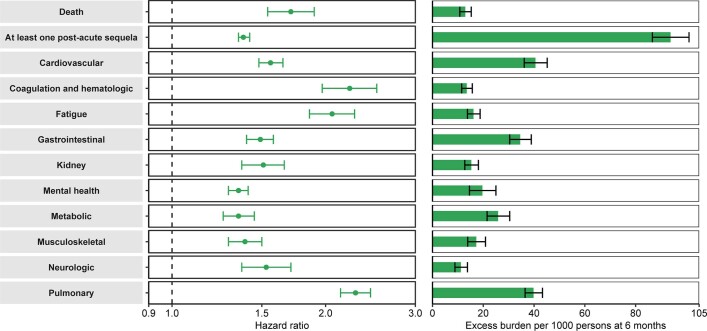
Risk and 6-month excess burden of post-acute sequelae in people with breakthrough SARS-CoV-2 infection compared to the historical control group. Risk and 6-month excess burden of death, at least one post-acute sequela, and post-acute sequelae by organ system are plotted. Incident outcomes were assessed from 30 days after the positive SARS-CoV-2 test to end of follow-up. Results are in comparison of breakthrough SARS-CoV-2 infection (n = 33,940) to the historical control group that consisted of those with no record of a positive SARS-CoV-2 test (n = 5,785,273). Adjusted hazard ratios (dots) and 95% confidence intervals (error bars) are presented, as are estimated excess burden (bars) and 95% confidence intervals (error bars). Burdens are presented per 1000 persons at 6 months of follow-up.

**Extended Data Fig. 3:**
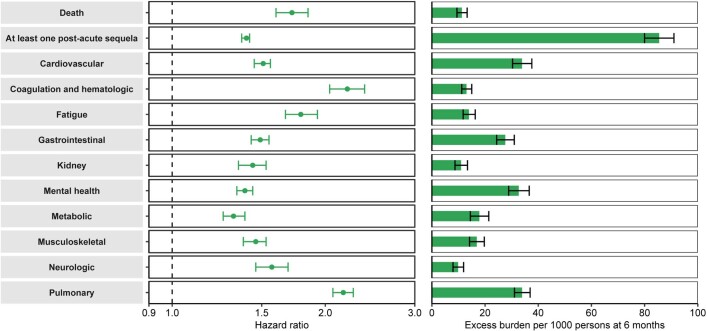
Risk and 6-month excess burden post-acute in people with breakthrough SARS-CoV-2 infection compared to the vaccinated control group. Risk and 6-month excess burden of death, at least one post-acute sequela, and post-acute sequelae by organ system are plotted. Incident outcomes were assessed from 30 days after the positive SARS-CoV-2 test to end of follow-up. Results are in comparison of breakthrough SARS-CoV-2 infection (n = 33,940) to the vaccinated control group that consisted of those with no record of a positive SARS-CoV-2 test (n = 2,566,369). Adjusted hazard ratios (dots) and 95% confidence intervals (error bars) are presented, as are estimated excess burden (bars) and 95% confidence intervals (error bars). Burdens are presented per 1000 persons at 6 months of follow-up.

The results were consistent in analyses considering a historical control group (*n* = 5,785,273) as the referent category (Extended Data Fig. 2 and Supplementary Table 7) and, separately, people who were vaccinated for SARS-CoV-2 and did not experience a BTI (*n* = 2,566,369) as another alternative control group (Extended Data Fig. 3 and Supplementary Table 8).

The risk of death was increased in the 30–90 days and also increased, but to a lesser extent, in the 90–180 days after a positive SARS-CoV-2 test (Supplementary Table 9). The risk of incident sequelae was increased in the 30–90 days after a positive SARS-CoV-2 test. In the period between 90 days and 180 days after testing positive, there was increased risk of both incident sequalae—albeit in lesser magnitude than the risk in days 30–90—and increased risk of recurrent or persistent sequalae (Supplementary Table 9).

**Extended Data Fig. 4:**
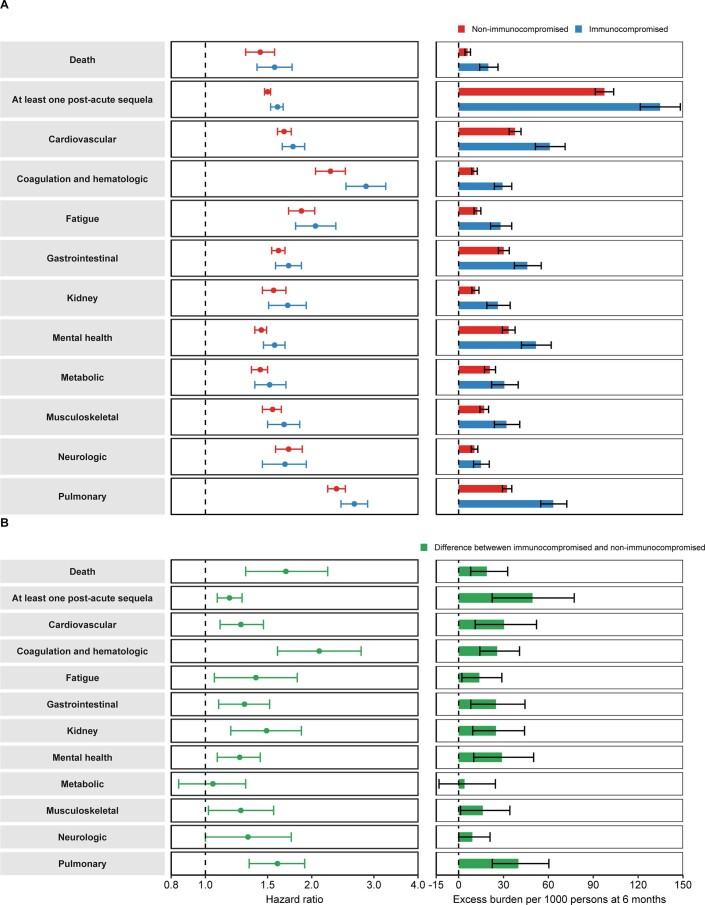
Risk and 6-month excess burden of post-acute sequelae in people with breakthrough SARS-CoV-2 infection by immunocompromised status. (A) Breakthrough SARS-CoV-2 infection compared to the contemporary control (n=4,983,491) in non-immunocompromised (n=27,431) and immunocompromised (n=6,509) individuals. (B) A within breakthrough SARS-CoV-2 infection comparison between immunocompromised (n=6,509) and non-immunocompromised (n=27,431) individuals. Incident outcomes were assessed from 30 days after the positive SARS-CoV-2 test to end of follow-up. Adjusted hazard ratios (dots) and 95% confidence intervals (error bars) are presented, as are estimated excess burden (bars) and 95% confidence intervals (error bars). Burdens are presented per 1000 persons at 6 months of follow-up.

Compared to the contemporary control group, there was increased risk of death, at least one PASC and organ involvement in people who were not immunocompromised before BTI (Extended Data Fig. 4a and Supplementary Table 10); the risks were generally higher in those who were immunocompromised before BTI (Extended Data Fig. 4a and Supplementary Table 10). Analyses of people with BTI showed that the risks of death, at least one PASC and organ system involvement were consistently higher in people who were immunocompromised versus those who were not before BTI (Extended Data Fig. 4b and Supplementary Table 10).

Of people with BTI, analyses by vaccine type suggested that there is no statistically significant difference in risk of post-acute death among the three SARS-CoV-2 vaccines (Pfizer-BioNTech (BNT162b2), Moderna (mRNA-1273) and Janssen (Johnson & Johnson) (Ad26.COV2.S)). Both BNT162b2 and mRNA-1273 were associated with decreased risk of at least one PASC: pulmonary and extrapulmonary organ involvement. There was no statistically significant difference in risk of any of these outcomes between BNT162b2 and mRNA-1273 (Supplementary Table 11).

### Post-acute sequelae in BTI by care setting of the acute phase of the disease

The demographic and health characteristics of people with BTI who were not hospitalized, who were hospitalized and who were admitted to ICU during the acute phase of the disease before and after weighting are provided in Supplementary Tables 12 and 13. Evaluation of standardized mean differences of baseline participant characteristics after the application of the weighting suggested good balance (Supplementary Fig. 2).

**Fig. 2:**
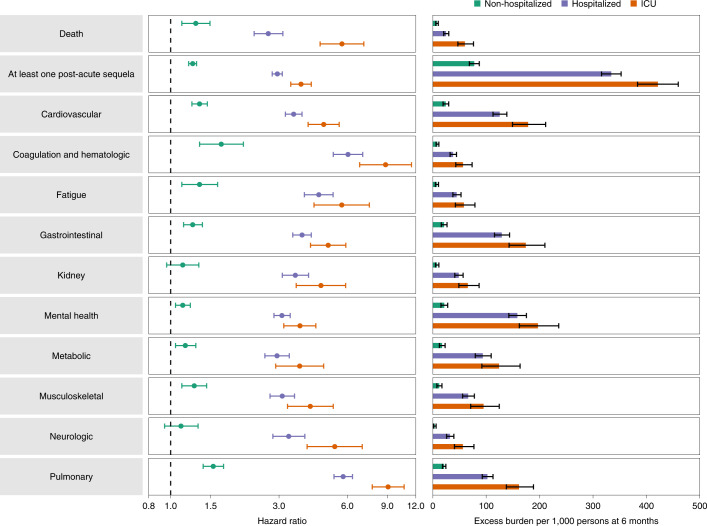
Risk and 6-month excess burden of post-acute sequelae in those with BTI by acute phase care setting. Risk and 6-month excess burden of death, at least one post-acute sequela and post-acute sequelae by organ system are plotted by care setting of the acute phase of the disease (not hospitalized, hospitalized and admitted to ICU). Incident outcomes were assessed from 30 days after the positive SARS-CoV-2 test to the end of follow-up. Results are in comparison of BTI (non-hospitalized *n* = 30,273; hospitalized *n* = 3,667; admitted to ICU *n* = 811) to the contemporary control group with no record of a positive SARS-CoV-2 test (*n* = 4,983,491). Adjusted HRs (dots) and 95% CIs (error bars) are presented, as are estimated excess burden (bars) and 95% CIs (error bars). Burdens are presented per 1,000 persons at 6 months of follow-up.

Compared to the control group of people without evidence of SARS-CoV-2 infection, people who were not hospitalized during the first 30 days of BTI exhibited an increased risk of death (HR = 1.29 (1.12, 1.49); burden of 7.77 (5.62, 10.24)); the risk was further increased in those who were hospitalized (HR = 2.69 (2.33, 3.12); burden of 24.79 (20.39, 29.86)) and was highest in those who were admitted to ICU (HR = 5.68 (4.55, 7.09); burden of 60.02 (46.85, 76.19)). The risk of having at least one post-acute sequela was evident in non-hospitalized people (HR = 1.25 (1.20, 1.30); burden of 77.60 (68.40, 87.04)), was further increased in those who were hospitalized (HR = 2.95 (2.80, 3.10); burden of 334.10 (315.90, 352.53)) and was highest in those admitted to ICU (HR = 3.75 (3.38, 4.16); burden of 421.39 (383.37, 459.56)) (Fig. 2 and Supplementary Table 14).

**Extended Data Fig. 5:**
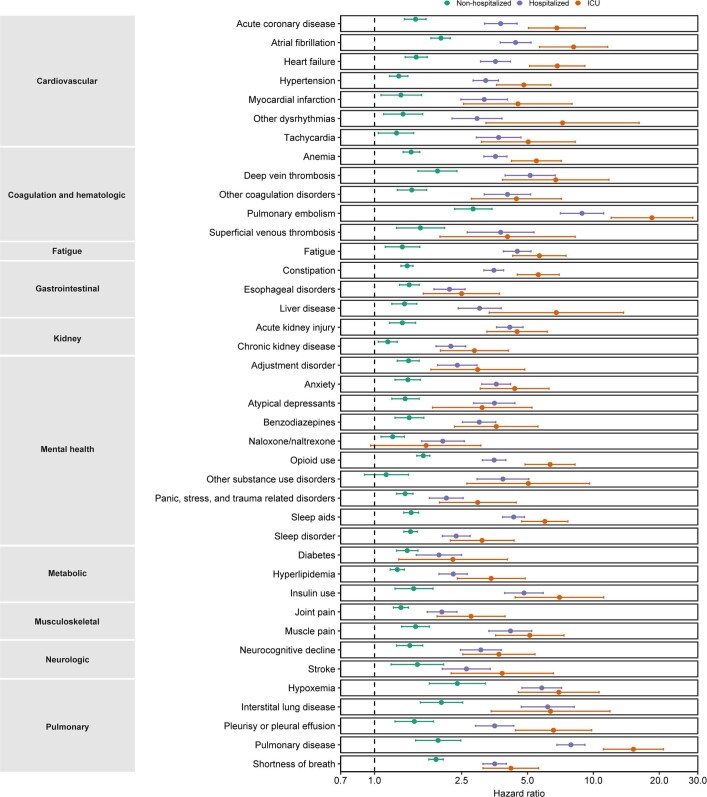
Risk of post-acute sequelae in people with breakthrough SARS-CoV-2 infection compared to the contemporary control group by acute phase care setting. Risks are plotted by care setting of the acute phase of the disease (not hospitalized, hospitalized, and admitted to the intensive care unit). Incident outcomes were assessed from 30 days after the positive SARS-CoV-2 test to end of follow-up. Results are in comparison of breakthrough SARS-CoV-2 infection (non-hospitalized n=30,273; hospitalized n=3,667; admitted to ICU n=811) to the contemporary control group that consisted of those with no record of a positive SARS-CoV-2 test (n=4,983,491). Adjusted hazard ratios (dots) and 95% confidence intervals (error bars) are presented. Outcomes that occurred in less than 10 participants in a group were excluded.

People who were not hospitalized exhibited small but significant increased risk of post-acute sequelae, including cardiovascular, coagulation and hematologic, gastrointestinal, mental health, metabolic, musculoskeletal and pulmonary disoders, as well as increased risk of fatigue (Fig. 2 and Supplementary Table 14). The risks were further increased in people who were hospitalized (Fig. 2 and Supplementary Table 14) and highest in those admitted to the ICU (Fig. 2 and Supplementary Table 14). Analyses of individual sequela are presented in Extended Data Fig. 5 and Supplementary Table 15.

### Post-acute sequelae in BTI versus SARS-CoV-2 infection without prior vaccination

To place the magnitude of risk of post-acute sequelae in people with BTI in broad context of post-acute COVID-19 manifestations, we developed a comparative approach to evaluate the risk of organ system involvement in people with BTI (*n* = 33,940) versus people with SARS-CoV-2 infection and no prior history of vaccination (*n* = 113,474) (Supplementary Tables 1 and 16). Assessment of standardized mean differences of baseline characteristics in the weighted cohorts suggested good balance (Supplementary Figs. 3 and 4 and Supplementary Tables 4 and 17).

**Fig. 3:**
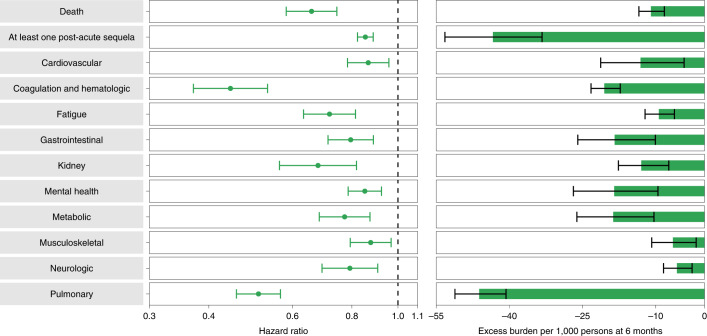
Risk and 6-month excess burden of post-acute sequelae in people with BTI compared to those with SARS-CoV-2 infection without prior vaccination. Risk and 6-month excess burden of death, at least one post-acute sequela and post-acute sequelae by organ system are plotted. Incident outcomes were assessed from 30 days after the positive SARS-CoV-2 infection test to the end of follow-up. Results are in comparison of BTI (*n* = 33,940) to those with SARS-CoV-2 infection without prior vaccination (*n* = 113,474). Adjusted HRs (dots) and 95% CIs (error bars) are presented, as are estimated excess burden (bars) and 95% CIs (error bars). Burdens are presented per 1,000 persons at 6 months of follow-up.

**Extended Data Fig. 6:**
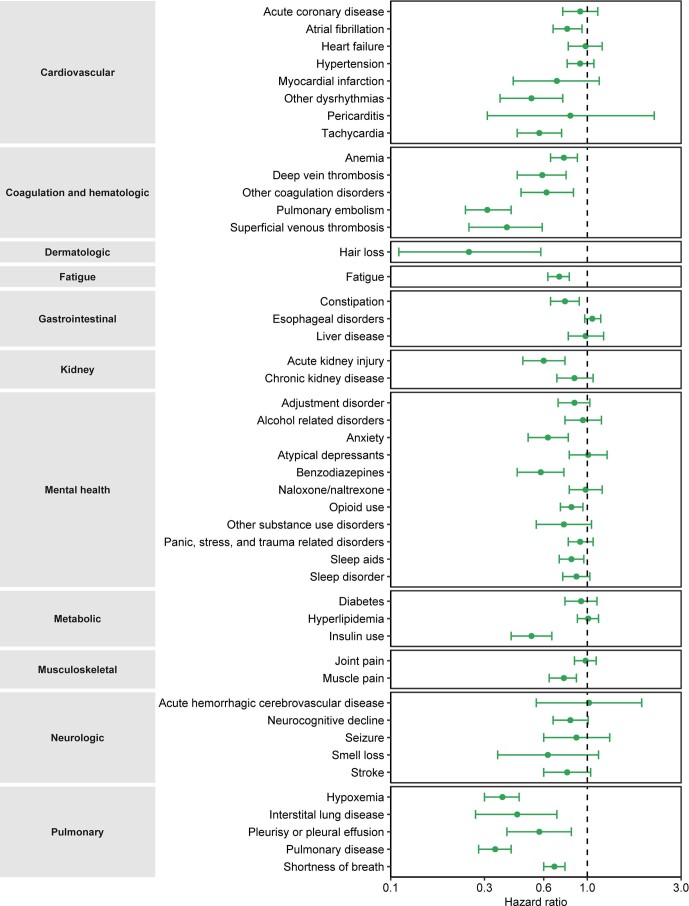
Risk of post-acute sequelae in people with breakthrough SARS-CoV-2 infection compared to those with SARS-CoV-2 infection without prior vaccination. Incident outcomes were assessed from 30 days after the positive SARS-CoV-2 test to end of follow-up. Results are in comparison of breakthrough SARS-CoV-2 infection (n=33,940) to those with SARS-CoV-2 infection without prior vaccination (n=113,474). Adjusted hazard ratios (dots) and 95% confidence intervals (error bars) are presented. Myocarditis (hazard ratio: 0.05; 95% confidence intervals: 0.01, 0.38) was not plotted to remove excess x-axis scaling.

People with BTI exhibited lower risk of death (HR = 0.66 (0.58, 0.74); burden of −10.99 (−13.45, −8.22); negative values denote reduced burden in BTI relative to SARS-CoV-2 infection) and lower risk of post-acute sequelae (HR = 0.85 (0.82, 0.89); burden of −43.38 (−53.22, −33.31)) compared to those with SARS-CoV-2 infection and no prior history of vaccination (Fig. 3 and Supplementary Table 18). Comparatively, the risk of post-acute sequelae in all the examined organ systems was lower in people with BTI versus those with SARS-CoV-2 infection without prior vaccination. BTI was associated with lower risk of 24 of the 47 sequelae examined compared to those with SARS-CoV-2 infection without prior vaccination (Extended Data Fig. 6 and Supplementary Table 19). The reduced risk was evident (albeit weak) in those who were immunocompromised and in those who were not immunocompromised (Supplementary Table 20).

**Fig. 4:**
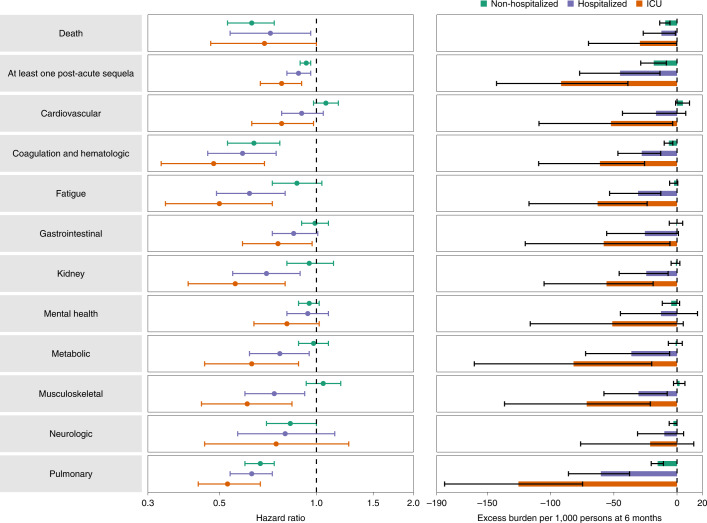
Risk and 6-month excess burden of post-acute sequelae in those with BTI compared to those with SARS-CoV-2 infection without prior vaccination by acute phase care setting. Risk and 6-month excess burden of death, at least one post-acute sequela and post-acute sequelae by organ system are plotted by care setting of the acute phase of the disease (not hospitalized, hospitalized and admitted to ICU). Incident outcomes were assessed from 30 days after the positive SARS-CoV-2 test to the end of follow-up. Results for a given care setting are in comparison of BTI (non-hospitalized *n* = 30,273; hospitalized *n* = 3,667; and admitted to ICU *n* = 811) to the SARS-CoV-2 infection without prior vaccination group (non-hospitalized *n* = 100,700; hospitalized *n* = 12,774; and admitted to ICU *n* = 2,982) with the same care setting during the acute phase of the disease. Adjusted HRs (dots) and 95% CIs (error bars) are presented, as are estimated excess burden (bars) and 95% CIs (error bars). Burdens are presented per 1,000 persons at 6 months of follow-up.

Analyses within each care setting suggested that the risk reduction in BTI versus SARS-CoV-2 infection on both the relative (HR) and absolute (burden) scale generally becomes increasingly more pronounced as the acuity of the care setting increased (from non-hospitalized to admitted to ICU) (Fig. 4 and Supplementary Table 21). BTI was associated with less risk of death and at least one PASC in all care settings. There was also a consistently reduced risk of hematologic and coagulation disorders and pulmonary disorders in BTI versus SARS-CoV-2 infection without prior vaccination across all care settings.

### Post-acute sequelae in people hospitalized with BTI versus seasonal influenza

We developed a comparative analysis to better understand how people hospitalized with BTI (*n* = 3,667) fare relative to those who are hospitalized with seasonal influenza (*n* = 14,337). Demographic and health characteristics before and after weighting are provided in Supplementary Tables 22 and 23. Examination of standardized mean differences of baseline characteristics after application of overlap weighting demonstrated good balance (Supplementary Fig. 5).

**Extended Data Fig. 7:**
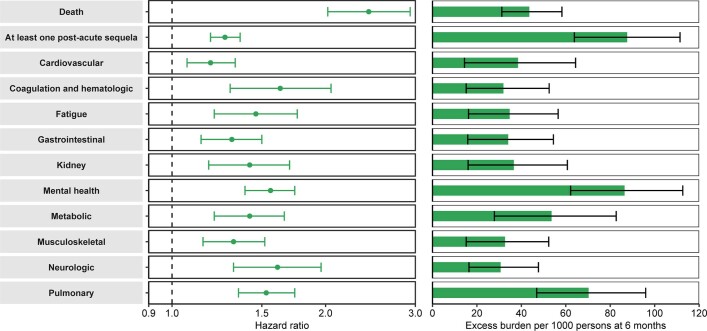
Risk and 6-month excess burden of post-acute sequelae in those hospitalized during the acute phase with breakthrough SARS-CoV-2 infection compared to seasonal influenza. Risk and 6-month excess burden of death, at least one post-acute sequela, and post-acute sequelae by organ system are plotted. Incident outcomes were assessed from 30 days after the positive SARS-CoV-2 test to end of follow-up. Results are in comparison of those hospitalized with breakthrough SARS-CoV-2 infection (n = 3,667) to those who were hospitalized with seasonal influenza (n = 14,337). Adjusted hazard ratios (dots) and 95% confidence intervals (error bars) are presented, as are estimated excess burden (bars) and 95% confidence intervals (error bars). Burdens are presented per 1000 persons at 6 months of follow-up.

Compared to people who were hospitalized with seasonal influenza, people with BTI who were hospitalized during the acute phase of the disease and survived the first 30 days exhibited an increased risk of death (HR = 2.43 (2.02, 2.93); burden of 43.58 (31.21, 58.26)) and increased risk of having at least one post-acute sequela (HR = 1.27 (1.19, 1.36); burden of 87.59 (63.83, 111.40)) (Extended Data Fig. 7 and Supplementary Table 24). People with BTI exhibited increased risk of sequelae in all the examined organ systems compared to those with seasonal influenza. Results of individual sequalae are presented in Supplementary Fig. 6 and Supplementary Table 25.

### Positive and negative outcome controls

To assess whether our approach reproduces established knowledge, we tested the association between SARS-CoV-2 infection without prior vaccination and the risk of fatigue (a cardinal post-acute sequela of COVID-19, where, based on prior evidence, we would expect a positive association). The results showed that, compared to the contemporary control group, people with SARS-CoV-2 infection and without prior vaccination exhibited increased risk of fatigue (HR = 2.79 (2.57, 303)) (Extended Data Table 1a).

To assess the putative presence of spurious associations, we tested the association between BTI and several negative outcome controls where there was no biologic plausibility or epidemiologic evidence that an association is expected. We used the same data sources, cohort building process, covariate selection approach (including predefined and algorithmically selected high-dimensional covariates), weighting method and interpretation of results. The results suggested no significant association between BTI and risk of any of the negative outcome controls (Extended Data Table 1a).

To further test the rigor of our approach, we tested as a pair of negative exposure controls receipt of influenza vaccination in odd-numbered (*n* = 605,453) versus even-numbered (*n* = 571,291) calendar days between 1 March 2020 and 15 January 2021. Examination of the associations of receipt of influenza vaccine on odd-numbered versus even-numbered calendar days and each outcome yielded non-significant results, consistent with our a priori expectations for a successful application of negative exposure controls (Extended Data Table 1b).

## Discussion

In this study of 33,940 people with BTI, 4,983,491 in the contemporary control, 5,785,273 in the historical control, 2,566,369 in the vaccinated control, 113,474 in the SARS-CoV-2 infection without prior vaccination group and 14,337 in the seasonal influenza group, we show that, compared to non-infected controls, people who survive the first 30 days of BTI exhibited increased risk of death and post-acute sequelae in the pulmonary and several extrapulmonary organ systems. The risks of death and post-acute sequelae were evident among non-hospitalized people, further increased among hospitalized people and highest among people who were admitted to ICU during the acute phase of the disease. Our comparative approach shows that risks of death and post-acute sequelae were lower in people with BTI versus people with SARS-CoV-2 infection without prior vaccination. Analyses of BTI versus SARS-CoV-2 infection without prior vaccination within the same care setting showed that this risk reduction was progressively more evident as care acuity of the acute phase of the disease increased from non-hospitalized to hospitalized and admitted to ICU and was consistently most pronounced for coagulation and pulmonary disorders. In comparative analyses among people who were hospitalized during the acute phase of the disease, those with BTI exhibited higher risks of death and post-acute sequelae than those with seasonal influenza. The constellation of findings shows that the burden of death and disease experienced by people with BTI is not trivial. Our comparative analyses provide a framework to better evaluate and contextually understand risks of the post-viral condition in people with BTI versus non-infected controls, versus SARS-CoV-2 infection without prior vaccination and versus seasonal influenza. The findings show that vaccination only partially reduces the risk of death and post-acute sequelae, suggesting that reliance on it as a sole mitigation strategy may not most optimally reduce the risk of the long-term health consequences of SARS-CoV-2 infection. Our results emphasize the need for continued optimization of primary prevention strategies of BTIs and will inform post-acute care approaches for people with BTI.

We examined the risk of death and post-acute sequelae in those with BTI versus several controls of people without evidence of SARS-CoV-2 infection, including (1) a contemporary control of people exposed to the same broader forces of the pandemic (lockdowns and economic, social and environmental stressors); (2) a historical control from a pre-pandemic era that represents a baseline unaffected by the disruptions of the pandemic; and (3) a vaccinated control group. The results show two key findings: (1) Long COVID, including increased risks of death and myriad post-acute sequelae in the pulmonary and extrapulmonary organ systems, also manifests in vaccinated individuals who experience a BTI; and (2) the range of post-acute sequelae in various organ systems in BTI does not appear to be different than COVID-19 without prior vaccination^1,4–12^. Our analyses of BTI versus SARS-CoV-2 infection without prior vaccination show that, comparatively, the magnitude of the risks of death and post-acute sequelae was lower in people with BTI versus those with SARS-CoV-2 infection who had not been previously vaccinated for it. These results show that, although vaccination may partially reduce the risks of post-acute death and disease, to most optimally reduce this burden requires continued emphasis on primary prevention of breakthrough SARS-CoV-2 infection as a goal of public health policy.

Although the absolute rates are smaller than in those with SARS-CoV-2 infection without prior vaccination, given the scale of the pandemic and the potential for breakthrough cases to continue to accumulate, the overall burden of death and disease after BTI will likely be substantial, will further add to the toll of this pandemic and will represent an additional strain on already overwhelmed health systems. In planning and development of health resources, governments and health systems should take into account the care needs of people with post-acute sequelae after BTI^13^.

Our analyses suggest that this risk reduction (of post-acute sequelae) was most pronounced in recipients of BNT162b2 and mRNA-1273 vaccines (compared to Ad26.COV2.S). Although these results recapitulate evidence of vaccine effectiveness in the acute phase of COVID-19, the mechanism or mechanisms underlying this carry-through effect of risk reduction from the acute to the post-acute phase of the disease is not entirely clear. One putative interpretation of these results is that vaccine-induced reduction in severity of the acute infection may then translate into less long-term risk of post-acute health outcomes. In other analyses, we also show that the reduced risk of post-acute sequelae in people with BTIs was partially eroded in people with immunocompromised status, suggesting a putative immune-related mechanism in the expression of post-acute sequelae that may be influenced by vaccination.

We also show that the risk of post-acute sequelae is higher in people with BTI than in people with seasonal influenza—a well-characterized respiratory viral illness. This extends previous evidence showing that the risk of post-acute sequelae in people with SARS-CoV-2 infection was higher than those with seasonal influenza and again emphasizes the importance of prevention of both SARS-CoV-2 infection and BTI^1^.

This study has several strengths. To our knowledge, it is the first large study to characterize the risks of post-acute sequelae of BTI at 6 months. We leveraged the vast national healthcare databases of the US Department of Veterans Affairs (the largest nationally integrated healthcare delivery system in the United States) to characterize the risk and 6-month burden of a comprehensive set of prespecified incident health outcomes in patients who survived the first 30 days of BTI versus several control groups (contemporary, historical and vaccinated controls). In addition to evaluating risk of BTI versus those with no evidence of SARS-CoV-2 infection in the overall cohort and by care setting of the acute phase of the disease (non-hospitalized, hospitalized and admitted to ICU), we also undertook a comparative evaluation of BTI versus SARS-CoV-2 infection in people who had not been previously vaccinated and, separately, BTI versus seasonal influenza. We used advanced statistical methodologies and adjusted through weighting for a battery of predefined covariates selected based on prior knowledge and algorithmically selected covariates from high-dimensional data domains, including diagnoses, prescription records and laboratory test results. We evaluated the rigor of our approach by testing positive and negative outcome controls to determine whether our approach would produce results consistent with pre-test expectations.

The study also has several limitations. The BTI and SARS-CoV-2 infection groups included only those who had a positive test for SARS-CoV-2 and did not include those who may have had an infection with SARS-CoV-2 but were not tested; however, if present, this will bias the estimates toward the null. Although the Veterans Affairs population is comprised of mostly men, it includes 8–10% women, which, across the groups in our study, included 1,300,744 female participants. Although we adjusted through the overlap weighting approach for a large battery of predefined and algorithmically selected covariates, and although our approach demonstrated good balance for more than 734 covariates (including all those that were available in the data but not included in the weighting process) from several data domains, including diagnoses, prescription medications and laboratory test results, and resulted in successful testing of positive outcome controls and negative outcome controls, we cannot completely rule out residual confounding. Our approach does not evaluate the severity of each outcome. Finally, the COVID-19 global pandemic is highly dynamic. As vaccine uptake continues to increase, as vaccine schedules continue to be optimized, as vaccine effectiveness wanes over time since vaccination, as booster vaccinations are deployed, as treatment strategies of the acute phase of COVID-19 continue to improve and as new variants of the virus emerge, it is likely that the epidemiology of BTI and its downstream sequelae may also change over time.

In sum, our findings provide evidence of increased risk of death and post-acute sequelae in people with BTI compared to controls with no evidence of SARS-CoV-2 infection; the risks were reduced in comparative analyses involving BTI versus SARS-CoV-2 infection without prior vaccination. Our results show that SARS-CoV-2 vaccination before infection only partially reduced the risk of death and post-acute sequelae. Measures for the prevention of breakthrough infections are needed to most optimally reduce the risk of the long-term health consequences of SARS-CoV-2 infection.

## Methods

All participants who were eligible for this study were enrolled; no a priori sample size analyses were conducted to guide enrollment. All analyses were observational, and investigators were aware of participant exposure and outcome status. A summary of the major design elements is presented in Supplementary Table 26, and an analytic flowchart is provided in Supplementary Fig. 7.

### Setting

Cohort participants were identified from the US Veterans Health Administration (VHA) electronic health databases. The VHA provides healthcare to discharged veterans of the US armed forces in a nationally integrated network of healthcare systems that includes more than 1,415 healthcare facilities. Veterans enrolled in the VHA have access to a comprehensive medical benefits package that includes outpatient services; preventive, primary and specialty care; mental health care; geriatric care; inpatient hospital care; extended long-term care; prescriptions; home healthcare; medical equipment; and prosthetics. The VHA healthcare databases are updated daily.

### Cohorts

We first identified users of the VHA who were alive on 1 January 2021 (*n* = 5,430,912). Use of the VHA was defined as having record of use of outpatient or inpatient service, receipt of medication or use of laboratory service with the VHA healthcare system in the 2 years prior (Supplementary Fig. 8). Among these, 163,024 participants had a record of a first positive SARS-CoV-2 test from 1 January 2021 to 31 October 2021, and 5,140,387 had no record of any positive SARS-CoV-2 test between 1 January 2020 and 1 December 2021. Participants were followed until 1 December 2021.

To construct a group of people with BTI, we selected, from those with a positive SARS-CoV-2 test (*n* = 163,024), those with a record of completion of an Ad26.COV2.S, mRNA-1273 or BNT162b2 vaccination before the date of their first positive SARS-CoV-2 test (*n* = 34,863). Completion of vaccination was defined following CDC guidelines at the 14th day after the second shot of the mRNA-1273 or BNT162b2 vaccination series or the 14th day after the first shot of the Ad26.COV2.S vaccination. Setting the date of first positive SARS-CoV-2 test as time zero (T_0_), we then selected those alive 30 days after T_0_, resulting in a cohort of 33,940 participants in the BTI group.

We then constructed several control groups; the rationale for each of these control groups is provided in Supplementary Fig. 9. To build a contemporary control group of people with no evidence of SARS-CoV-2 infection, we then used the 5,140,387 users of the VHA who had no record of a SARS-CoV-2-positive test. Among these participants, we randomly assigned a T_0_ to each participant in the group on the basis of the distribution of the T_0_ dates in those with BTI. We finally selected those who were alive 30 days after their T_0_ (*n* = 4,983,491). The contemporary control group represents contemporaneous users of the VHA who were subject to the broader forces of the pandemic but did not contract SARS-CoV-2 infection. Of these, the 2,566,369 who had record of a SARS-CoV-2 vaccination before their T_0_ served as a vaccinated control group. The vaccinated control group represents contemporaneous users of the VHA who share the characteristic of being vaccinated with the breakthrough group and have a major distinction in that they did not contract SARS-CoV-2 infection subsequent to their vaccination.

To build an alternate control group during a period of time where participants were not subject to the influence of the pandemic, we identified users of the VHA who were alive on 1 January 2018 (*n* = 6,084,973) and who had no history of a positive SARS-CoV-2 test (*n* = 5,938,519). After randomly assigning a T_0_ in 2018 on the basis of the distribution of the calendar dates of T_0_ in those with BTI, 5,785,273 were alive 30 days after T_0_. Participants were followed until 1 December 2018. This group served as the historical control group.

To build the group of people with SARS-CoV-2 infection and without prior vaccination as a means of investigating the effect of prior vaccination on the risk of post-acute sequalae, we identified, from the 163,024 people with a first positive SARS-CoV-2 test from 1 January 2021 to 31 October 2021, 118,185 who had no record of any SARS-CoV-2 vaccination up through 30 days after first positive SARS-CoV-2 test (T_0_). We then selected the 113,474 who were alive 30 days after T_0_ to comprise the group of people with SARS-CoV-2 infection and no prior vaccination.

Finally, to compare post-acute sequelae of those hospitalized with BTI during the acute phase of the illness to those hospitalized with seasonal influenza, we separately identified 15,160 VHA users hospitalized with positive seasonal influenza test 5 days before or 30 days after the test between 1 October 2016 and 29 February 2020. We set the date of the positive seasonal influenza test as T_0_. To ensure no overlap with the BTI group, participants who had no record of a positive SARS-CoV-2 test were then selected (*n* = 14,431). From these, we selected 14,337 who were alive 30 days after their T_0_ to constitute the seasonal influenza group. Duration of follow-up was randomly assigned on the basis of follow-up in the BTI group.

### Data sources

Data used in this study were obtained from the VHA Corporate Data Warehouse (CDW). Within CDW, the patient data domain provided information on demographic characteristics; the outpatient encounters domain and inpatient encounters domain provided information on health characteristics, including data on timing and location of interactions with the healthcare system, diagnoses and procedures; the pharmacy and barcode medication administration domains provided medication records; and the laboratory results domain provided laboratory test information in both outpatient and inpatient settings^5,6^. The COVID-19 Shared Data Resource provided information on SARS-CoV-2 test results and SARS-CoV-2 vaccination status. The 2019 Area Deprivation Index (ADI) at each cohort participant residential address was used as a contextual measure of socioeconomic disadvantage^14^.

### Post-acute sequelae

We prespecified a set of outcomes based on prior evidence on the post-acute sequelae of SARS-CoV-2 infection—also referred to as Long COVID^4–12^. Outcomes were defined using validated definitions leveraging information from several data domains, including diagnoses, prescription medications and laboratory test results, at the time of first record of occurrence in the data^5,6,15–21^. Incident post-acute sequelae were examined in a cohort with no record of the health condition in the 2 years before T_0_. We additionally examined outcomes of death and having at least one of post-acute sequelae that was defined at the time of the first incident prespecified post-acute sequelae in each participant.

Additionally, we defined a set of outcomes where we aggregated the prespecified post-acute sequelae, where applicable, by organ system. These included cardiovascular disorders, coagulation and hematologic disorders, fatigue, gastrointestinal disorders, kidney disorders, mental health disorders, metabolic disorders, musculoskeletal disorders, neurologic disorders and pulmonary disorders. All outcomes were assessed starting from 30 days after T_0_.

### Covariates

We included a set of predefined covariates based on prior knowledge^4–12,19,22–26^ and algorithmically selected covariates. Predefined covariates included demographic information (age, race and sex); contextual information (ADI); measures of the intensity of healthcare interaction in the 2 years before T_0_, including the number of outpatient visits, the number of inpatient visits, the number of unique medications the participant received a prescription for and the number of routine blood panels that were performed; and prior history of receiving an influenza vaccination. We also included smoking status as a covariate. Health characteristics included prior history of anxiety, cancer, cardiovascular disease, cerebrovascular disease, chronic kidney disease, peripheral artery disease, dementia, depression, type 2 diabetes mellitus and chronic obstructive pulmonary disease, and measures of estimated glomerular filtration rate, systolic and diastolic blood pressure, and body mass index (BMI). We also included, as measures of spatiotemporal differences, the calendar week of enrollment and geographic region of receipt of care defined by Veterans Integrated Services Networks (VISN).

In consideration of the dynamicity of the pandemic, for analyses that compared BTI, SARS-CoV-2 infection without prior vaccination and the contemporary control, additional covariates included SARS-CoV-2 testing capacity, SARS-CoV-2 positivity rates, hospital system capacity (the total number of inpatient hospital beds) and inpatient bed occupancy rates (the percentage of hospital beds that were occupied) as well as a measure of the proportions of SARS-CoV-2 variants by Health and Human Services region^26^. These measures were ascertained for each participant in the week before cohort enrollment at the location of the healthcare system at which they received care. In analyses of the vaccinated control, we additionally included calendar week of first vaccination shot. All continuous covariates were treated as natural cubic splines unless heavily skewed toward zero.

In addition to the predefined covariates, we leveraged the high dimensionality of VA data where we developed and deployed a high-dimensional variable selection algorithm to identify covariates that may potentially confound the examined associations^27^. Using classifications from the Clinical Classifications Software Refined version 2021.1, available from the Healthcare Cost and Utilization Project sponsored by the Agency for Healthcare Research and Quality, more than 70,000 ICD-10 diagnoses codes in the year before T_0_ for each participant were classified into 540 diagnostic categories^28–30^. Using the VA drug classification system, 3,425 different medications were classified into 543 medication classes^31,32^. Finally, laboratory results from 38 different laboratory measurements were classified into 62 laboratory test abnormalities, defined by being above or below the corresponding reference ranges, on the basis of the recorded Logical Observation Identifiers Names and Codes. Of the high-dimensional variables that occurred at least 100 times in participants in each group (up to 821), we selected the top 100 variables with the highest relative risk for differences in group membership for inclusion in models.

### Statistical analysis

Mean (standard deviation) and frequency (percentage) of select characteristics are reported in the BTI group, SARS-CoV-2-infected group without prior vaccination, the contemporary control group, the historical control group, the vaccinated control group and the seasonal influenza group, where appropriate. Characteristics of those with BTI by hospitalization status are additionally presented. Vaccination characteristics for those with BTI are reported as well as BTI rates per 1,000 persons at 6 months for those vaccinated from 1 January to 31 October 2021.

To balance baseline characteristics, including predefined and high-dimensional variables across comparison groups, we applied an overlap weighting approach in our analyses. In brief, logistic regressions were constructed for probability of group membership of the groups being compared, using the predefined and high-dimensional covariates as independent variables, in separate subcohorts with no prior history of the outcome being examined, estimating propensity scores of the probability of group assignment^33,34^. In consideration of variability in duration of potential follow-up, calendar week of enrollment was included to balance length of follow-up between cohorts (uncensored duration of follow-up was included for comparison versus seasonal influenza). Propensity scores were then used in construction of the overlap weights whose application achieved similar baseline characteristic distributions across groups while providing higher weights to those with baseline characteristics more similar to those in other groups. Weights were then applied to Cox survival models to estimate HRs, where follow-up started 30 days after the date of testing positive. Standard errors were estimated by applying the robust sandwich variance estimator method. Covariate balance among all predefined and high-dimensional variables were assessed for each model through the standardized mean difference, where a difference <0.1 was taken as evidence of balance. We estimated the incidence rate difference (referred to as excess burden) between groups per 1,000 participants at 6 months after the start of follow-up based on the difference in survival probability in the relevant groups.

We first examined the risk and excess burden of individual post-acute sequelae, post-acute sequelae by organ system, at least one post-acute sequela and death between the BTI group, those with SARS-CoV-2 infection without prior vaccination and the contemporary control. We then compared risks of the BTI group with the historical control and, separately, with the vaccinated control.

Further analyses were conducted to better understand the risk in BTI versus the contemporary control. To investigate risk of post-acute sequalae before and after 90 days of follow-up, we conducted analyses that examined risk during the first 30–90 days, and during the 90–180 days, after T_0_. Examination of the risk from the 90–180 days was done overall, for incident outcomes during this period (where there was no record of the outcome during the 30–90-day period) and for recurrent or persistent outcomes during this period (where there was a prior record of the outcome during the 30–90-day period). We then comparatively evaluated the risks between the BTI and contemporary control based on their immunocompromised status, where immunocompromised status was defined (according to the CDC definition) by a history of organ transplantation, advanced kidney disease (an estimated glomerular filtration rate of less than 15 ml/min/1.73 m^2^ or end-stage renal disease), cancer, HIV or conditions with prescriptions of more than 30-day use of corticosteroids or immunosuppressants, including systemic lupus erythematosus and rheumatoid arthritis. We lastly compared the risks and burden of death, at least one PASC, pulmonary disorders and extrapulmonary disorders within those with BTI by type of vaccination received.

We then examined the risk and excess burden associated with BTI by care setting of the acute phase of the disease. Risks were estimated for individual sequelae and risks and excess burden of organ system involvement, at least one post-acute sequela and death in those with a BTI who were not hospitalized, who were hospitalized and who were admitted to ICU during the 5 days before and 30 days after their positive SARS-CoV-2 test compared to the contemporary control group.

We additionally examined differences in risk and burden between BTI and SARS-CoV-2 infection without prior vaccination by severity of the acute phase of the disease (non-hospitalized, hospitalized and admitted to ICU).

Finally, we compared the risks and excess burden of individual post-acute sequelae, post-acute sequelae by organ system, at least one post-acute sequela and death between those hospitalized with BTI and those hospitalized with seasonal influenza.

### Positive and negative controls

We examined, as positive outcome controls, the risks of fatigue in those with SARS-CoV-2 infection without prior vaccination compared to the contemporary and historical control groups as a means of testing whether our approach would reproduce established knowledge^8–12^.

The application of negative outcome control may help detect both suspected and unsuspected sources of spurious biases. We, therefore, tested comparing BTI to the contemporary and historical controls, the risk of atopic dermatitis, accidental poisoning, accidental injury, fitting of a hearing aid or contact lenses, ingrown toenail and scarring as negative outcome controls—where no prior knowledge suggests that an association is expected. Additionally, we tested a pair of negative-exposure controls; we expected that receipt of the influenza vaccine on odd-numbered (*n* = 605,453) versus even-numbered (*n* = 571,291) calendar days between 1 March 2020 and 15 January 2021 would be associated with similar risks of the outcomes examined in our analyses. The successful testing of positive outcome controls, negative outcome controls and negative exposure controls may lessen concerns about biases related to study design, covariate selection, analytic approach, outcome ascertainment, unmeasured confounding and other potential sources of latent biases^35,36^.

All analyses were two-sided. In all analyses, a 95% CI that excluded unity was considered evidence of statistical significance. All analyses were conducted in SAS Enterprise Guide 8.2, and all figures were generated in R version 4.0.4. This study was approved by the VA St. Louis Health Care System Institutional Review Board (protocol no. 1606333).

### Reporting summary

Further information on research design is available in the Nature Research Reporting Summary linked to this article.

## Online content

Any methods, additional references, Nature Research reporting summaries, source data, extended data, supplementary information, acknowledgements, peer review information; details of author contributions and competing interests; and statements of data and code availability are available at 10.1038/s41591-022-01840-0.

### Supplementary information

Supplementary Information Supplementary TOC and Supplementary Figs. 1–9

Reporting Summary

Supplementary Data 1 This file contains all supplementary data tables (Supplementary Data Tables 1–26).

## Data Availability

The data that support the findings of this study are available from the US Department of Veterans Affairs. VA data are made freely available to researchers behind the VA firewall with an approved VA study protocol. For more information, visit https://www.virec.research.va.gov or contact the VA Information Resource Center at VIReC@va.gov.

## Extended data

**Extended Data Table 1:** Positive and negative controls

(A)			
Outcome	Contemporary control HR (95% CI)		Historical control HR (95% CI)
**Positive outcome control**			
Fatigue	2.79 (2.57, 3.03)		2.93 (2.68, 3.21)
**Negative outcome controls**			
Atopic dermatitis	1.01 (0.89, 1.15)		1.06 (0.96, 1.17)
Accidental poisoning	0.98 (0.88, 1.09)		1.01 (0.90, 1.13)
Accidental injury	1.01 (0.89, 1.13)		1.00 (0.90, 1.11)
Fitting of a hearing aid or contact lenses	1.00 (0.93, 1.06)		1.00 (0.93, 1.07)
Ingrown toenail	0.99 (0.96, 1.03)		1.00 (0.96, 1.03)
Scar	1.01 (0.90, 1.14)		0.99 (0.88, 1.12)
Models were weighted for age, race, ADI, the number in the year before testing positive of outpatient visits, inpatient visits and unique medication prescriptions, routine blood chemistry panels, prior history of receiving an influenza vaccination, smoking status, anxiety, cancer, cardiovascular disease, cerebrovascular disease, chronic kidney disease, dementia, depression, type 2 diabetes mellitus, chronic obstructive pulmonary disease, peripheral artery disease, systolic blood pressure, BMI, geographic region of VISN and calendar week of enrollment as well as algorithmically selected high-dimensional variables. Comparisons with the contemporary control additionally weighted for hospital system average hospital bed occupancy, total hospital bed capacity, positivity rate of tests, number of tests and Health and Human Services region SARS-CoV-2 variant proportion in the week before enrollment.			
**(B)**			
**Outcome**		**HR (95% CI)**	
Death		1.00 (0.98, 1.02)	
At least one post-acute sequela		1.00 (0.99, 1.01)	
Cardiovascular		1.00 (0.99, 1.01)	
Coagulation and hematologic		1.01 (0.98, 1.03)	
Fatigue		1.00 (0.98, 1.02)	
Gastrointestinal		1.01 (0.99, 1.02)	
Kidney		1.00 (0.98, 1.02)	
Mental health		1.00 (0.99, 1.01)	
Metabolic		1.00 (0.99, 1.01)	
Musculoskeletal		0.99 (0.97, 1.01)	
Neurologic		1.01 (0.99, 1.02)	
Pulmonary		1.00 (0.98, 1.02)	
Models were weighted for age, race, ADI, the number in the year before testing positive of outpatient visits, inpatient visits and unique medication prescriptions, routine blood chemistry panels, BMI, smoking status, anxiety, cancer, cardiovascular disease, cerebrovascular disease, chronic kidney disease, dementia, depression, type 2 diabetes mellitus, chronic obstructive pulmonary disease, peripheral artery disease, systolic blood pressure, diastolic blood pressure, estimated glomerular filtration rate, geographic region of VISN and duration of follow-up as well as algorithmically selected high-dimensional variables.			

(A): evaluation of a positive outcome control in those with SARS-CoV-2 infection without prior vaccination and negative outcome controls in those with breakthrough SARS-CoV-2 infection compared to the contemporary and historical control groups. (B): evaluation of the association of a negative exposure control of receipt of seasonal influenza vaccination on odd calendar days compared to even calendar days with risk of outcomes.

## Code availability

The analytic code is available at https://github.com/BcBowe3/Breakthrough.
