# Supplementary material for: Long COVID after breakthrough SARS-CoV-2 infection
Source: Nat Med. 2022 May 25;28(7):1461–7. doi: 10.1038/s41591-022-01840-0 (PMC9307472; doi:10.1038/s41591-022-01840-0)

nature portfolio

https://doi.org/10.1038/s41591-022-01840-0

Supplementary information

Long COVID after breakthrough SARS-CoV-2 infection

In the format provided by the authors and unedited

Supplementary Information

Long COVID after breakthrough SARS-CoV-2 infection

Table of Contents

Table of Contents	Page
**Supplementary Figures**	
**Supplementary Figure 1:** Standardized mean differences between those with breakthrough SARS-CoV-2 infection and the contemporary control group before and after weighting by organ system.	4
**Supplementary Figure 2:** Standardized mean differences between those with breakthrough SARS-CoV-2 infection that were non-hospitalized, hospitalized, and admitted to the intensive care unit during the acute phase of the disease and the contemporary control group before and after weighting by organ system.	5
**Supplementary Figure 3:** Standardized mean differences between those with breakthrough SARS-CoV-2 infection and those with SARS-COV-2 infection without prior vaccination by organ system.	6
**Supplementary Figure 4:** Standardized mean differences between those with breakthrough SARS-CoV-2 infection and those with SARS-COV-2 infection without prior vaccination that were non-hospitalized, hospitalized, and admitted to the ICU during the acute phase of the disease by organ system.	7
**Supplementary Figure 5:** Standardized mean differences between those with breakthrough SARS-CoV-2 infection hospitalized during the acute phase of the illness and those hospitalized with seasonal influenza before and after weighting by organ system.	8
**Supplementary Figure 6:** Risk of post-acute sequelae in people who were hospitalized during the acute phase of breakthrough SARS-CoV-2 infection compared to those hospitalized with seasonal influenza	9
**Supplementary Figure 7:** Analytic flowchart	10
**Supplementary Figure 8:** Cohort flowchart	11
**Supplementary Figure 9:** Rationale and interpretation of the comparative analyses	12
**Supplementary Tables**	
**Supplementary Table 1:** Demographic and health characteristics of those with breakthrough SARS-CoV-2 infection, SARS-COV-2 infection without prior vaccination, the contemporary control group, the historical control group, and the vaccinated control group before weighting.	Excel sheet
**Supplementary Table 2:** Demographic and health characteristics of those with breakthrough SARS-CoV-2 infection and SARS-COV-2 infection without prior vaccination by immunocompromised status before weighting.	Excel sheet
**Supplementary Table 3:** Demographic and health characteristics of those with breakthrough SARS-CoV-2 infection by vaccine status before weighting.	Excel sheet
**Supplementary Table 4:** Characteristics and standardized mean differences of those with breakthrough SARS-CoV-2 infection, SARS-COV-2 infection without prior vaccination, and the contemporary control group after weighting.	Excel sheet
**Supplementary Table 5:** Risk and 6-month burden of death, at least one post-acute sequela, and post-acute sequelae by organ system in breakthrough SARS-CoV-2 infection compared to the contemporary control group.	Excel sheet
**Supplementary Table 6:** Risk of post-acute sequelae in breakthrough SARS-CoV-2 infection compared to the contemporary control group.	Excel sheet
**Supplementary Table 7:** Risk and 6-month burden of death, at least one post-acute sequela, and post-acute sequelae by organ system in breakthrough SARS-CoV-2 infection compared to the historical control group.	Excel sheet
**Supplementary Table 8:** Risk and 6-month burden of death, at least one post-acute sequela, and post-acute sequelae by organ system in breakthrough SARS-CoV-2 infection compared to the vaccinated control group.	Excel sheet

**Supplementary Table 9:** Risk and burden of death, at least one post-acute sequela, and post-acute sequelae by organ system in breakthrough SARS-CoV-2 infection compared to the contemporary control group in the first 30-90, and 91-180, days of follow-up.	Excel sheet
**Supplementary Table 10:** Risk and 6-month burden of death, at least one post-acute sequela, and post-acute sequelae by organ system in those with breakthrough SARS-CoV-2 infection by immunocompromised status.	Excel sheet
**Supplementary Table 11:** Risk and 6-month burden of death, at least one post-acute sequela, and post-acute sequelae by organ system in breakthrough SARS-CoV-2 infection by vaccination type.	Excel sheet
**Supplementary Table 12:** Demographic and health characteristics of those with breakthrough SARS-CoV-2 infection by care setting of the acute phase before weighting before weighting.	Excel sheet
**Supplementary Table 13:** Characteristics and standardized mean differences of those with breakthrough SARS-CoV-2 infection by care setting of the acute phase of the disease compared to the contemporary control group after weighting.	Excel sheet
**Supplementary Table 14:** Risk and 6-month burden of death, at least one post-acute sequela, and post-acute sequelae by organ system in those with breakthrough SARS-CoV-2 infection compared to the contemporary control group by care setting of the acute phase of the disease.	Excel sheet
**Supplementary Table 15:** Risk of post-acute sequelae in those with breakthrough SARS-CoV-2 infection compared to the contemporary control group by care setting of the acute phase of the disease.	Excel sheet
**Supplementary Table 16:** Demographic and health characteristics of those with SARS-COV-2 infection without prior vaccination by care setting of the acute phase before weighting.	Excel sheet
**Supplementary Table 17:** Characteristics and standardized mean differences of those with breakthrough SARS-CoV-2 infection and SARS-COV-2 infection without prior vaccination by care setting of the acute phase of the disease after weighting.	Excel sheet
**Supplementary Table 18:** Risk and 6-month burden of death, at least one post-acute sequela, and post-acute sequelae by organ system in breakthrough SARS-CoV-2 infection compared to those with SARS-COV-2 infection without prior vaccination.	Excel sheet
**Supplementary Table 19:** Risk of post-acute sequelae in breakthrough SARS-CoV-2 infection compared to those with SARS-COV-2 infection without prior vaccination.	Excel sheet
**Supplementary Table 20:** Risk and 6-month burden of death, at least one post-acute sequela, and post-acute sequelae by organ system in those with breakthrough SARS-CoV-2 infection compared to those with SARS-COV-2 infection without prior vaccination by immunocompromised status.	Excel sheet
**Supplementary Table 21:** Risk and 6-month burden of death, at least one post-acute sequela, and post-acute sequelae by organ system in those with breakthrough SARS-CoV-2 infection compared to those with SARS-COV-2 infection without prior vaccination by care setting of the acute phase of the disease.	Excel sheet
**Supplementary Table 22:** Demographic and health characteristics of those hospitalized with breakthrough SARS-CoV-2 infection and seasonal influenza before weighting.	Excel sheet
**Supplementary Table 23:** Characteristics and standardized mean differences of those with breakthrough SARS-CoV-2 infection hospitalized during the acute phase of the disease compared to those hospitalized with seasonal influenza after weighting.	Excel sheet
**Supplementary Table 24:** Risk and 6-month burden of death, at least one post-acute sequela, and post-acute sequelae by organ system in breakthrough SARS-CoV-2 infection hospitalized during the acute phase compared to those hospitalized with seasonal influenza.	Excel sheet
**Supplementary Table 25:** Risk of post-acute sequelae in breakthrough SARS-CoV-2 infection hospitalized during the acute phase compared to those hospitalized with seasonal influenza.	Excel sheet

**Supplementary Table 26:** Summary table of study design elements	Excel sheet

Supplementary Figure 1: Standardized mean differences between those with breakthrough SARS-CoV-2 infection and the contemporary control group before and after weighting by organ system.

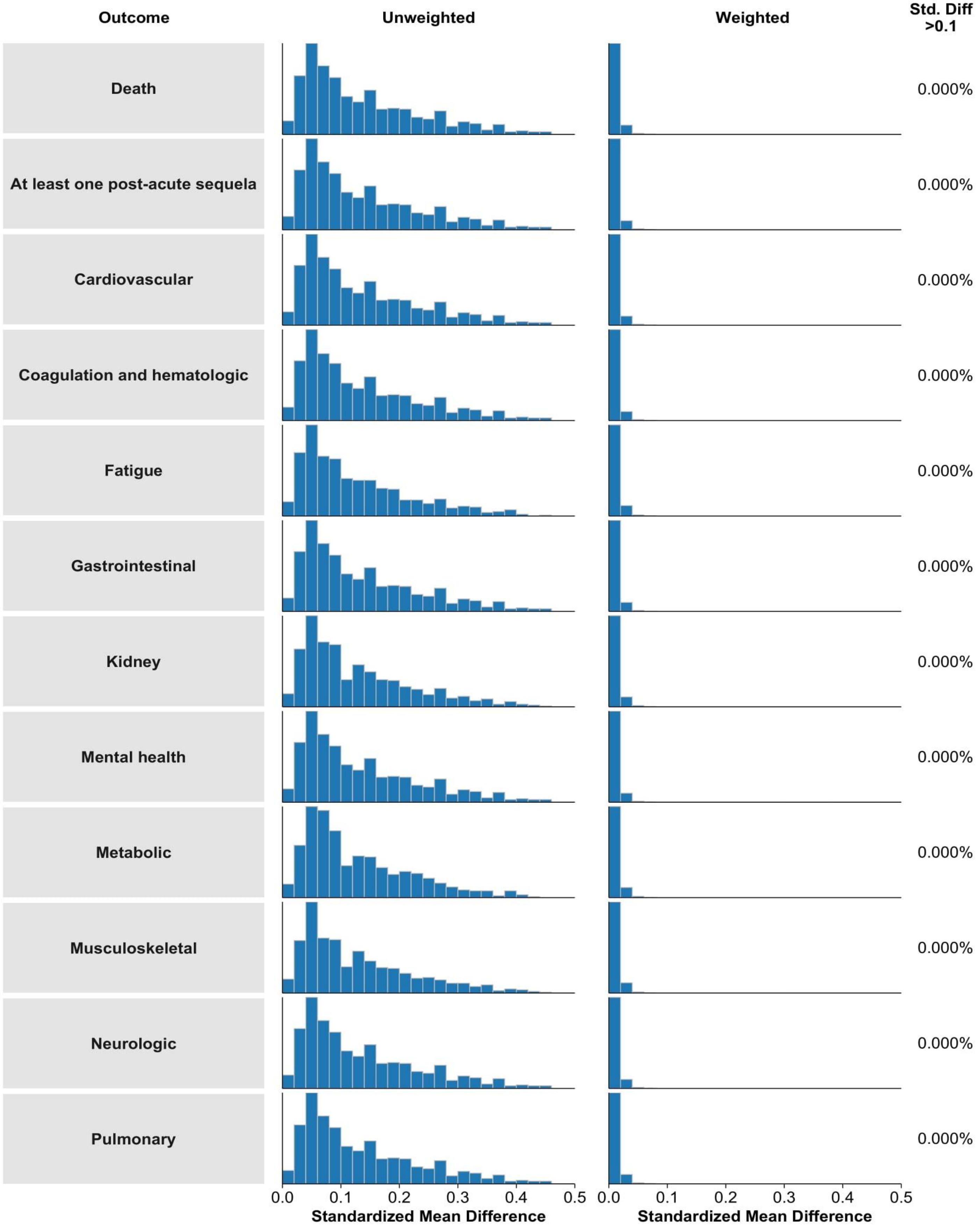

Supplementary Figure 2: Standardized mean differences between those with breakthrough SARS-CoV-2 infection that were non-hospitalized, hospitalized, and admitted to the intensive care unit during the acute phase of the disease and the contemporary control group before and after weighting by organ system.

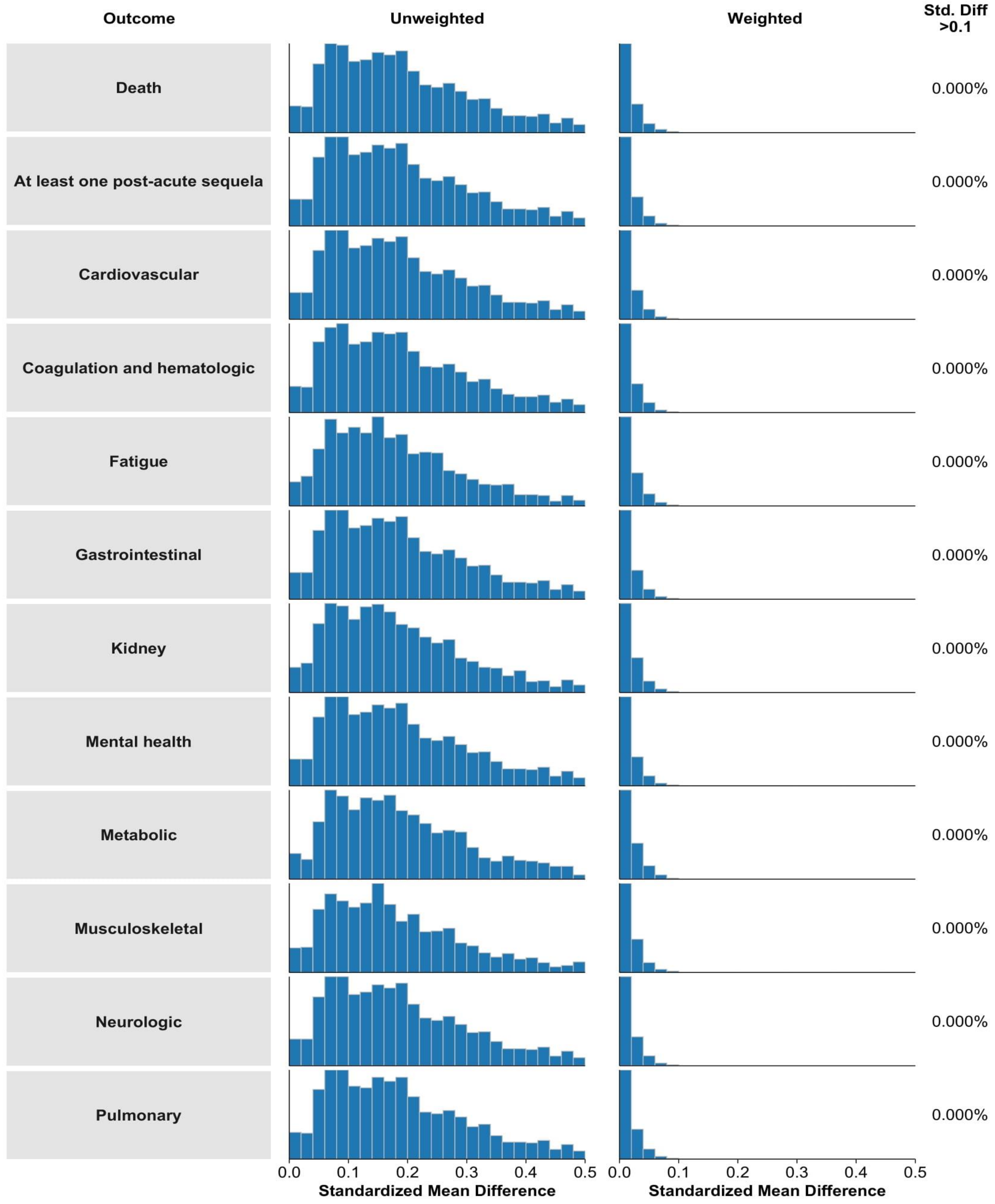

Supplementary Figure 3: Standardized mean differences between those with breakthrough SARS-CoV-2 infection and those with SARS-COV-2 infection without prior vaccination by organ system.

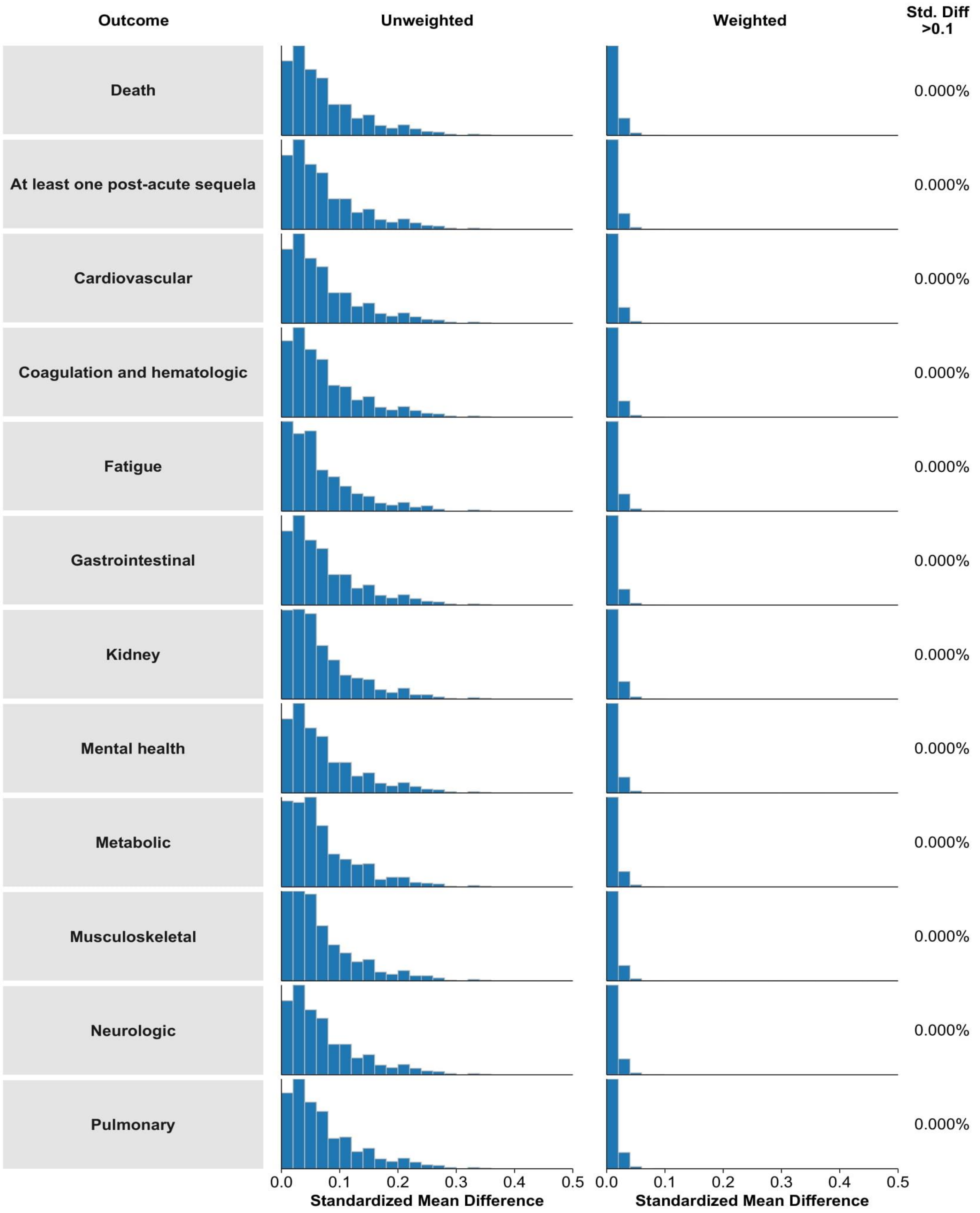

Supplementary Figure 4: Standardized mean differences between those with breakthrough SARS-CoV-2 infection and those with SARS-COV-2 infection without prior vaccination that were non-hospitalized, hospitalized, and admitted to the ICU during the acute phase of the disease by organ system.

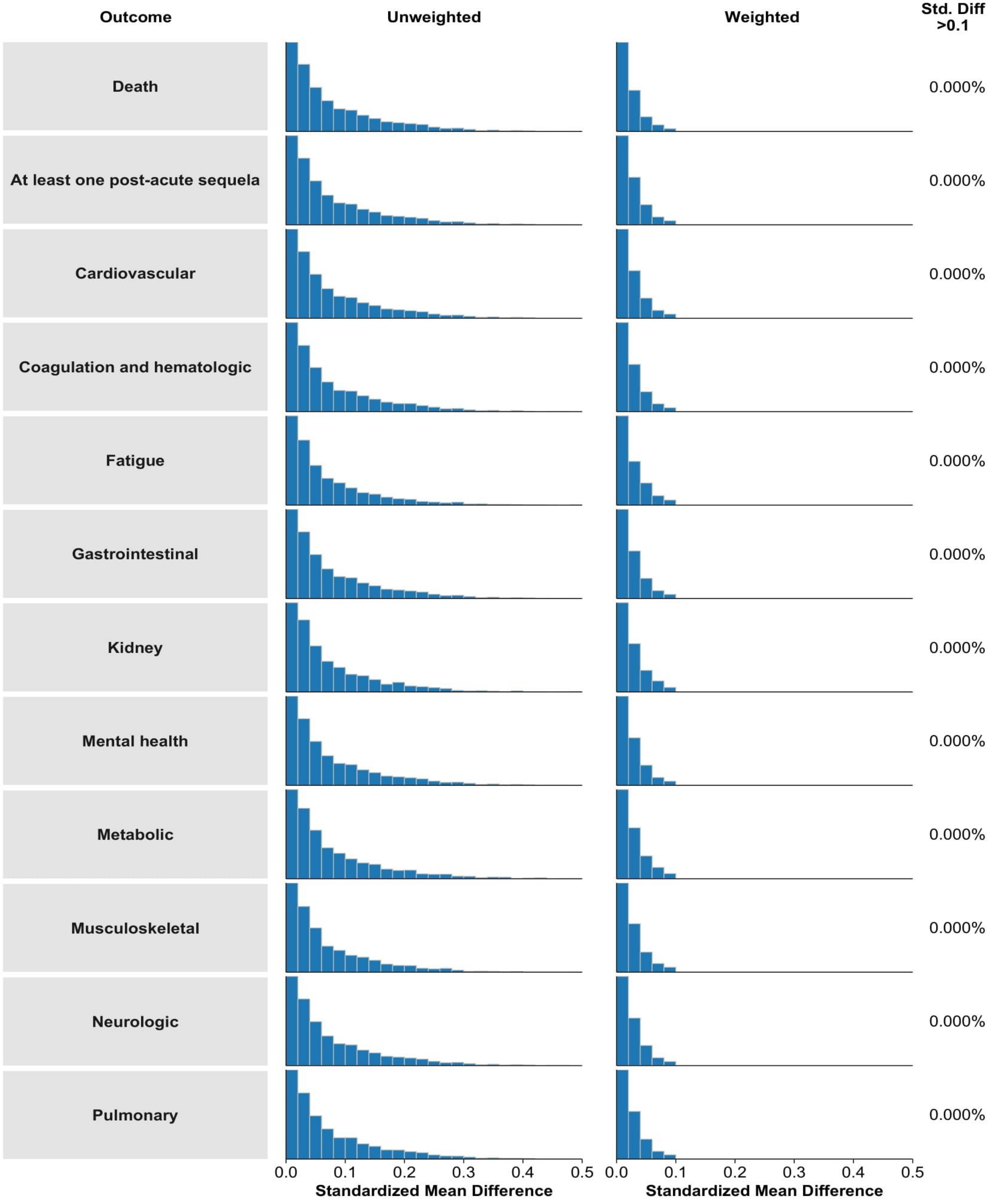

Supplementary Figure 5: Standardized mean differences between those with breakthrough SARS-CoV-2 infection hospitalized during the acute phase of the illness and those hospitalized with seasonal influenza before and after weighting by organ system.

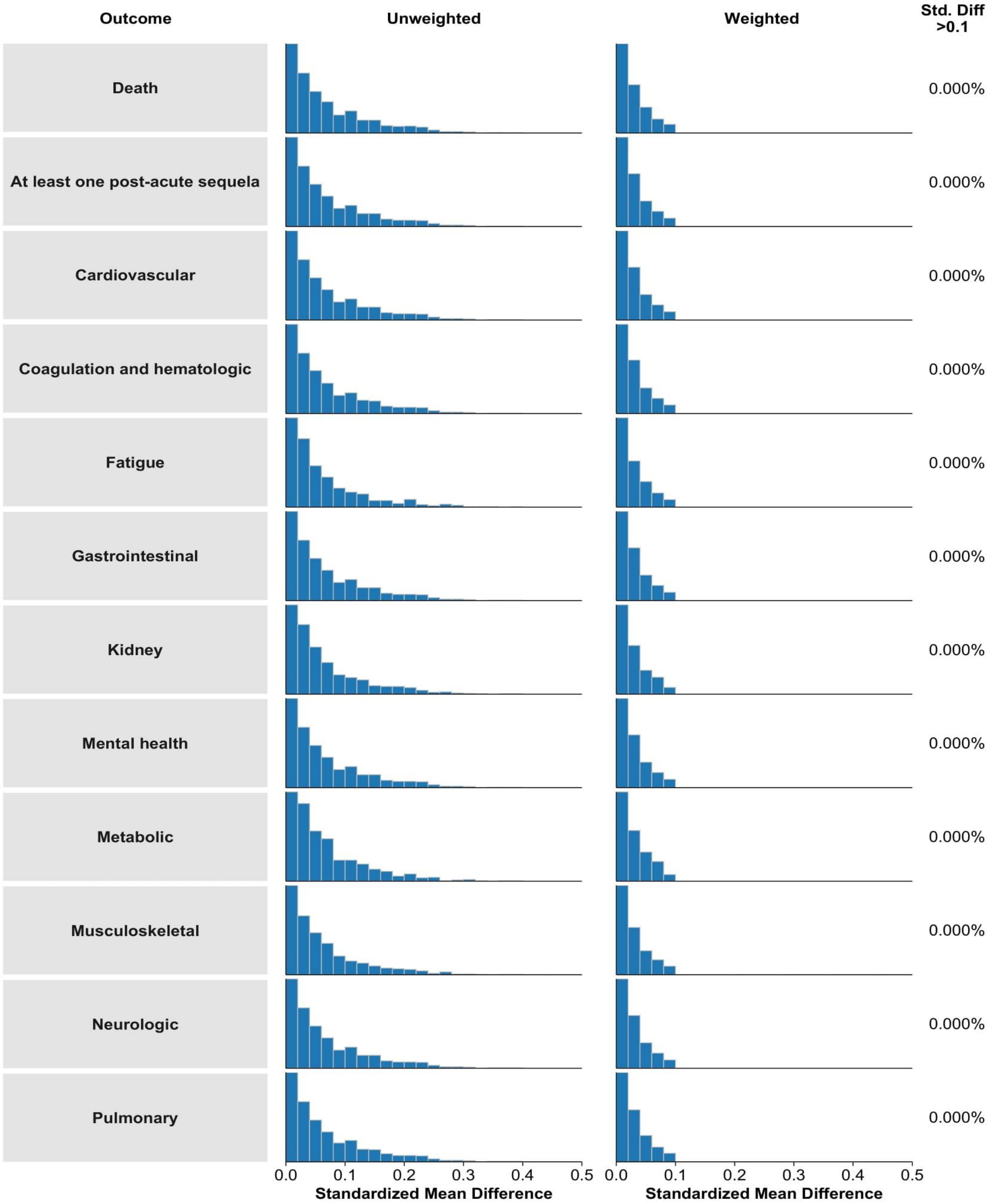

Supplementary Figure 6: Risk of post-acute sequelae in people who were hospitalized during the acute phase of breakthrough SARS-CoV-2 infection compared to those hospitalized with seasonal influenza. Incident outcomes were assessed from 30 days after the positive SARS-COV-2 infection test to end of follow-up. Results are in comparison of those hospitalized with breakthrough SARS-CoV-2 infection (n=3,667) to those hospitalized with seasonal influenza (n=14,337). Adjusted hazard ratios (dots) and 95% confidence intervals (error bars) are presented. Outcomes that occurred in less than 10 participants in a group were excluded.

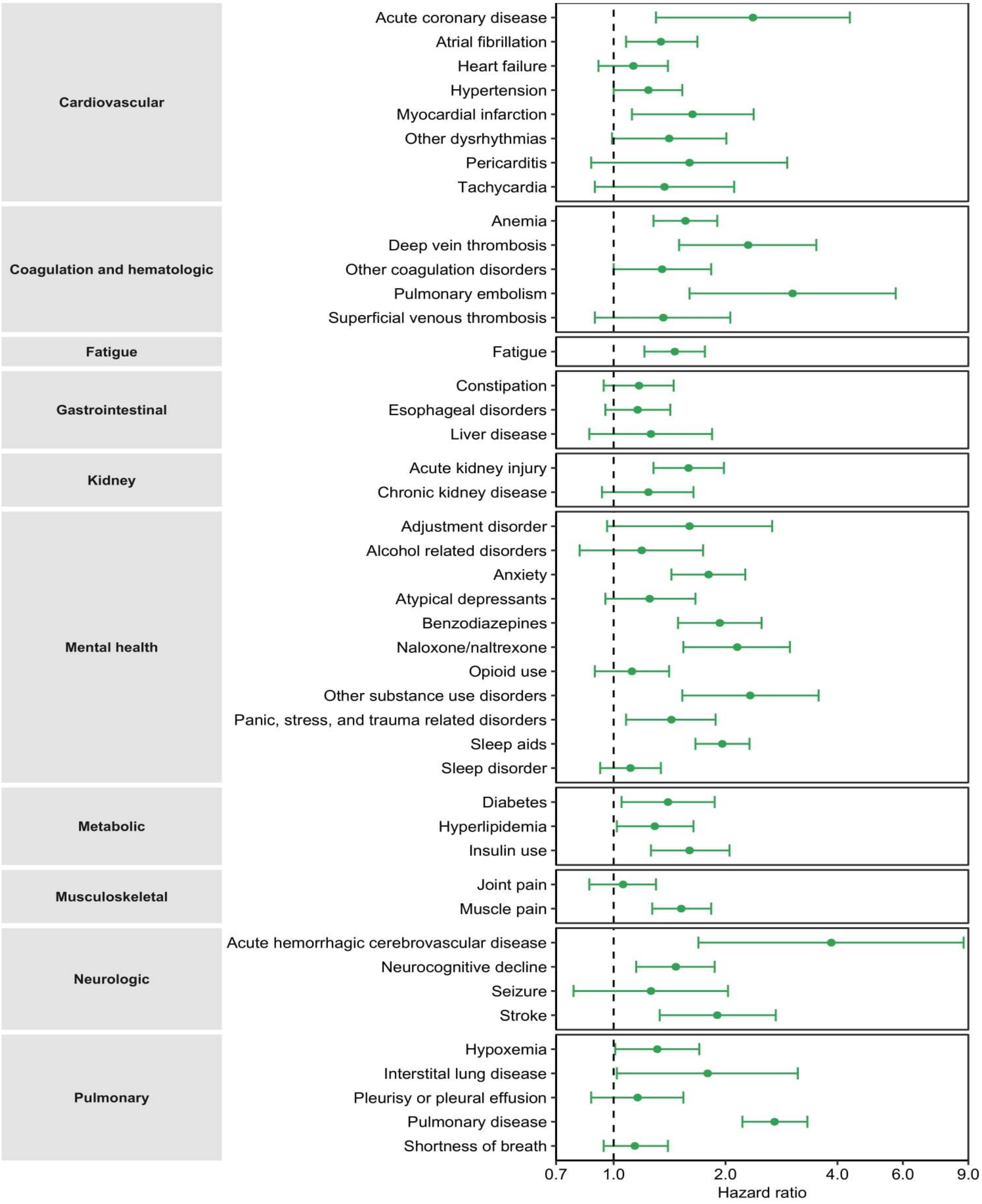

Supplementary Figure 7: Analytic flowchart.

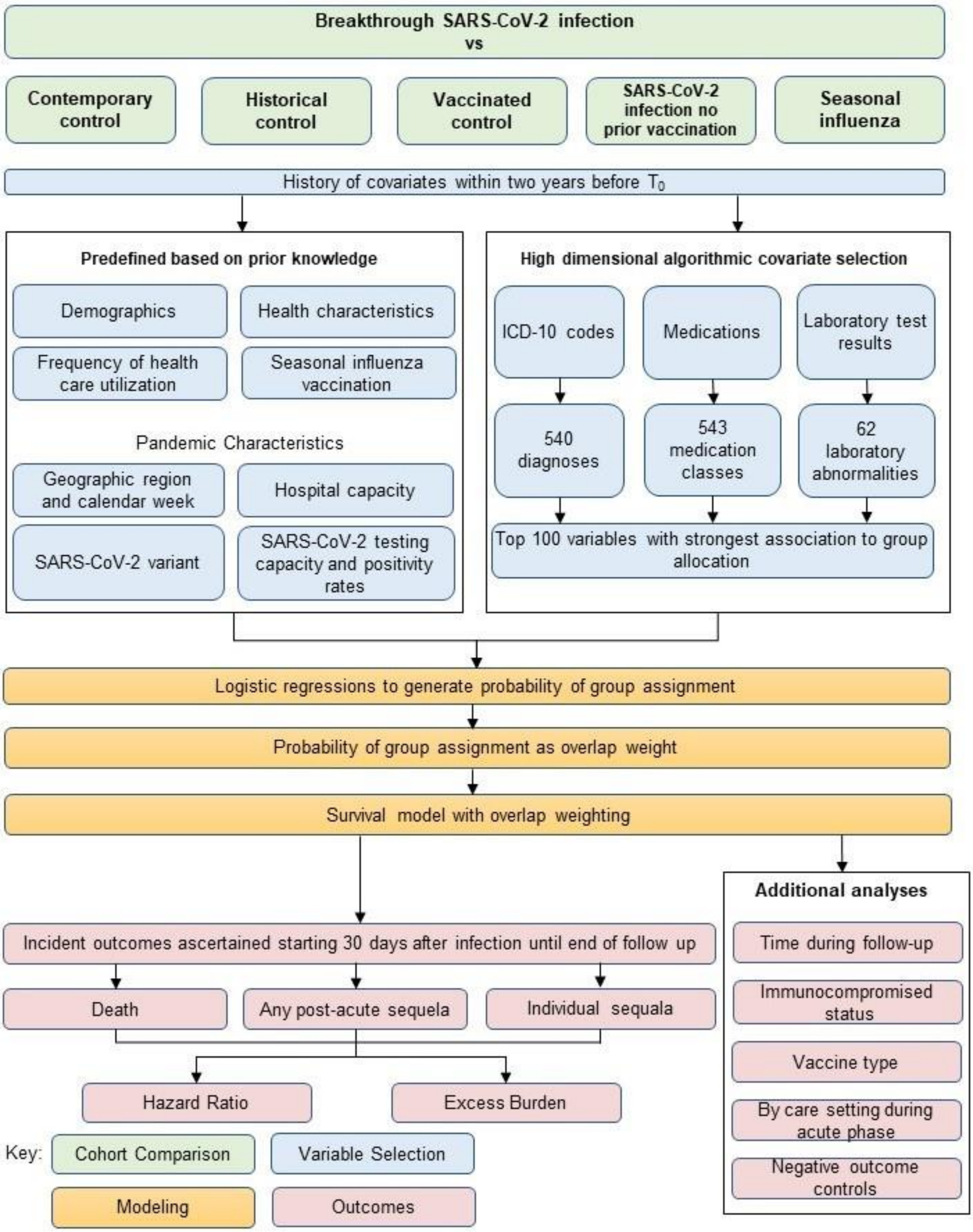

Supplementary Figure 8: Cohort flowchart.

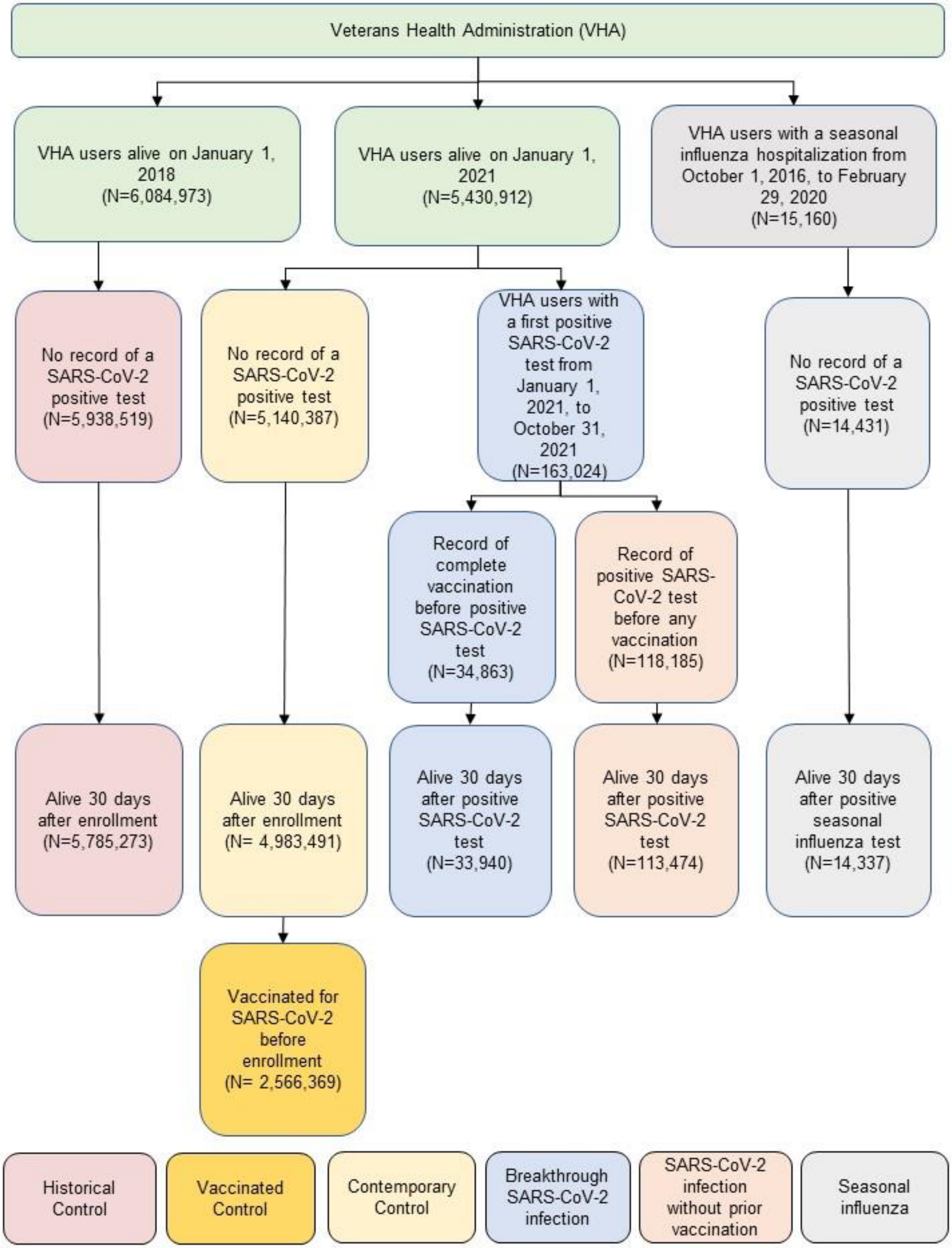

Supplementary Figure 9: Rationale and interpretation of the comparative analyses

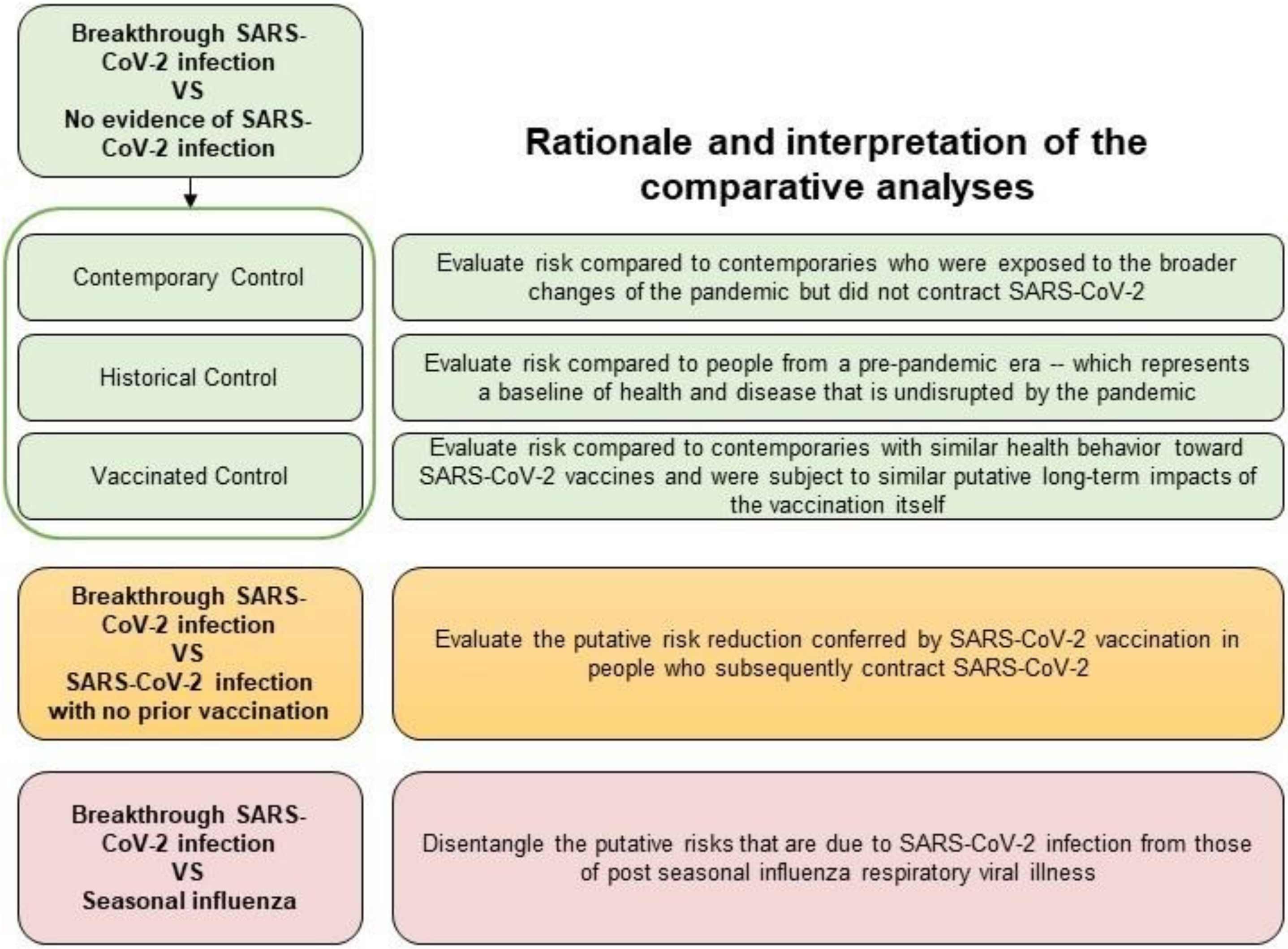

Supplement: Supplementary file 1 — Supplementary TOC and Supplementary Figs. 1–9 [file 41591_2022_1840_MOESM1_ESM.pdf]
